# Molecular Mechanisms Contributing to Resistance to Tyrosine Kinase-Targeted Therapy for Non-Small Cell Lung Cancer

**DOI:** 10.3969/j.issn.2095-3941.2012.01.003

**Published:** 2012-03

**Authors:** Fariz Nurwidya, Akiko Murakami, Fumiyuki Takahashi, Kazuhisa Takahashi

**Affiliations:** Department of Respiratory Medicine, Juntendo University School of Medicine, Tokyo 113-8421, Japan

**Keywords:** carcinoma, non-small-cell lung, mutation, drug resistance

## Abstract

One of the most important pathways in non-small cell lung cancer (NSCLC) is the epidermal growth factor receptor (EGFR) pathway. This pathway affects several crucial processes in tumor development and progression, including tumor cell proliferation, apoptosis regulation, angiogenesis, and metastatic invasion. Targeting EGFR is currently being intensely explored. We are witnessing the development of a number of potential molecular-inhibiting treatments for application in clinical oncology. In the last decade, the tyrosine kinase (TK) domain of the EGFR was identified in NSCLC patients, and it has responded very well with a dramatic clinical improvement to TK inhibitors such are gefitinib and erlotinib. Unfortunately, there were primary and/or secondary resistance to these treatments, as shown by clinical trials. Subsequent molecular biology studies provided some explanations for the drug resistance phenomenon. The molecular mechanisms of resistance need to be clarified. An in-depth understanding of these targeted-therapy resistance may help us explore new strategies for overcoming or reversing the resistance to these inhibitors for the future of NSCLC treatment.

## Introduction

In recent years, the leading cause of cancer-related deaths has been lung cancer, accounting for one third of all cancer deaths worldwide. In the United States, lung cancer has the highest incidence of mortality among all cancer types in both males and females ^[^^1^^]^. Based on pathological findings, lung cancer has been classified into two, namely, non-small cell lung cancer (NSCLC; 80% incidence) and small-cell lung cancer (SCLC; 20% incidence). NSCLC is sub-classified as squamous cell carcinoma, adenosquamous cell carcinoma, large cell carcinoma, adenocarcinoma, and others ^[^^2^^]^. Adenocarcinoma, squamous carcinoma, and large cell carcinoma are some of the most common lung cancers. Over the last several years, significant advances in the fields of medical oncology, biomarkers, tumor biology, and radiodiagnostics have expanded our understanding of lung adenocarcinoma. Consequently, the need for revising the current classification system has arisen. The revision should involve an integrated multidisciplinary platform. Nevertheless, the light microscopic evaluation of pathological materials still plays a crucial role in the diagnosis and sub-classification of lung adenocarcinoma in the absence of molecular, immunohistochemical, or histochemical testing.

The epidermal growth factor (EGF) receptor (EGFR) is one of the most important discoveries in the field of medical oncology. This review discusses the EGFR, the targeted therapies inhibiting this receptor, and the occurrence of resistance.

## EGFR

Growth factor studies began in 1952 when Rita Levi-Montalcini discovered the nerve growth factor (NGF) in the laboratory of Viktor Hamburger ^[^^3^^]^. This growth factor was purified by Levi-Montalcini and Stanley Cohen in 1957 from snake venom and mouse salivary gland extracts ^[^^3^^]^. Five years later, Cohen discovered the epidermal growth factor (EGF) as it stimulated the proliferation of epithelial cells ^[^^3^^]^. For these discoveries, Levi-Montalcini and Cohen received the Nobel Prize in Physiology or Medicine in 1986.

EGFR overexpression is observed in about 62% of NSCLC cases, and is associated with unfavorable prognosis ^[^^4^^]^. EGFR is a member of the receptor tyrosine kinase (TK) family that includes ERBB2, ERBB3, and ERBB4 ^[^^5^^]^. These receptors consist of an extracellular ligand-binding domain, a transmembrane domain, and an intracellular domain ^[^^5^^]^. The intracellular TK activity of EGFR increases upon binding to various cognate ligands, such as EGF, which leads to the homodimerization of two EGFRs or the heterodimerization of EGFR with other family members. The activation of receptor TKs leads to the autophosphorylation of the intracellular domain of EGFR, resulting in the activation of the Ras/mitogen-activated protein kinase (MAPK) pathway, phosphatidylinositol 3-kinases (PI3K)/Akt pathway, as well as signal transducers and activators of transcription signaling pathways.

## TK Inhibitor (TKI)

The specific inhibition of the EGFR TK by small molecules, such as imatinib for chronic myelogeneous leukemia, started the targeted therapy era in cancer treatment. Subsequently, gefitinib and erlotinib strengthened the importance of targeted therapy. Gefitinib was the first drug to become clinically available for the treatment of NSCLC. Japan approved gefitinib for the first time in 2002^[^^6^^]^. The Food and Drug Administration of the United States also approved gefitinib in 2003 and erlotinib in 2004 for the treatment of NSCLC ^[^^7^^]^. Favorable clinical responses to targeted therapy are more common in East Asian than European and U.S. American patients, in women than men, in non-smokers than smokers, and in patients with adenocarcinoma than those with other histologic subtypes ^[^^8^^]^.

## EGFR Mutation

Mutations in the kinase domain of EGFR are usually referred to as activating mutations, indicating that these mutations result in increased kinase activity of the receptor ^[^^4^^]^. However, not all mutated EGFR are necessarily constitutively active. The kinase domain is located from exon 18 to 21 ^[^^4^^]^. There are EGFR mutations that render NSCLC sensitive to gefitinib. The types of EGFR mutations in gefitinib-responding patients worldwide are quite similar ^[^^8^^]^. Such mutations include multiple overlapping deletion mutations of exon 19 in 45% of patients, point mutations in exon 21 in 40% of patients (predominantly L858R), and point or insertion mutations in exons 18 to 21 in the remaining 15% of patients ^[^^8^^]^.

Clinical trials on gefitinib efficacy for advanced NSCLC with EGFR mutations in Japan were summarized by Morita et al. ^[^^9^^]^ A total of 148 NSCLC patients with EGFR mutations from seven eligible trials were identified. The overall response rate to gefitinib was 76.4% [95% confidence interval (95%CI), 69.5-83.2]. The median progression-free survival and overall survival were 9.7 (95%CI, 8.2-11.1) and 24.3 (95%CI, 19.8-28.2) months, respectively. Good performance status and chemotherapy-naïve status were significantly associated with a longer progression-free survival or overall survival. Among the 148 patients, 87 received gefitinib as the first-line therapy, whereas 61 received systemic chemotherapy before gefitinib treatment. The median progression-free survival after the start of first-line therapy was significantly longer in the gefitinib-first group than in the chemotherapy-first group (10.7 *vs.* 6.0 months; *P*<0.001). No significant difference was found in the median overall survival between the 2 groups (27.7 *vs.* 25.7 months; *P*=0.782) ^[^^9^^]^.

## TKI Drug Resistance

**Figure 1:**
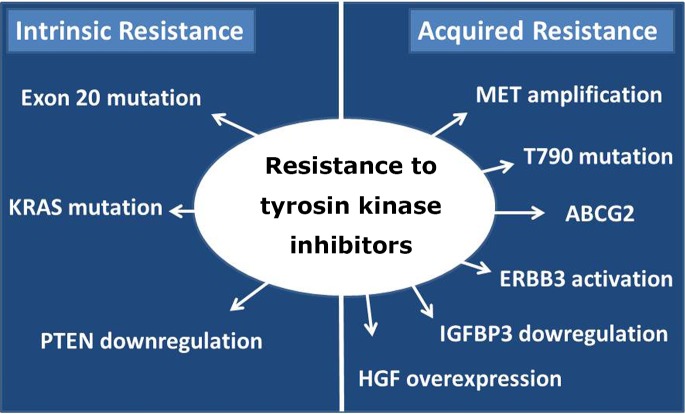
Two types of resistance to TKIs: intrinsic and acquired.

The EGFR mutation status aids in the identification of the subgroup of NSCLC patients who can benefit the most from TKI targeted therapy. Nevertheless, not all EGFR mutations are correlated with sensitivity to gefitinib and erlotinib. We can distinguish two types of resistance to TKI, namely, intrinsic and acquired resistance (**Figure 1**).

### Intrinsic resistance

Intrinsic resistance (also known as primary resistance) is defined as drug resistance in a tumor that is initially refractory to EGFR TK inhibitor treatment ^[^^10^^]^. Some molecular mechanisms have been classified as primary resistance, such as exon 20 mutations, KRAS mutation, as well as phosphatase and tensin homolog (PTEN) downregulation.

#### Exon 20 mutation

Wu et al. ^[^^11^^]^ examined the clinical features of lung cancer patients with the exon 20 mutations, which cause NSCLC patients to be less responsive to gefitinib treatment, although there are variabilities among different individuals. NSCLC patients with exon 20 mutations showed a gefitinib response rate of 25%, far lower than that of patients with exon 19 deletions and L858R mutations ^[^^11^^]^. Exon 20 mutations are relatively rare. This finding suggests that other mechanisms may be involved in EGFR-TKI intrinsic resistance ^[^^12^^]^.

#### KRAS mutation

Another somatic mutation that has also been associated with primary resistance to EGFR-TKI is the mutation in exon 2 (codon 12–13) of KRAS, which encodes a GTPase downstream of EGFR ^[^^13^^]^. The signaling downstream of mutated KRAS acts in parallel with EGFR by activating the PI3K and MAPK signaling pathways, thereby enabling the activation of these pathways independent of EGFR ^[^^7^^]^. Pao et al. ^[^^14^^]^ determined whether KRAS mutations can be used to predict response to gefitinib or erlotinib. They examined 60 lung adenocarcinomas defined as sensitive or refractory to gefitinib or erlotinib for mutations in EGFR and KRAS. Their result indicated that KRAS was associated with lack of sensitivity to both drugs ^[^^14^^]^.

#### Downregulation of PTEN expression

PTEN is a lipid phosphatase that removes the phosphate from the D3 position of phosphatidylinositol 3,4,5- triphosphate, thereby directly antagonizing the action of PI3Ks ^[^^15^^]^. Hence, PTEN is a critical negative regulator of the ubiquitous PI3K pathway that transduces signals regulating growth, proliferation, and survival.

Resistance to gefitinib and erlotinib has also been identified in PC-9 cells (human lung adenocarcinoma cells) with silenced PTEN expression using small interfering RNA specific for PTEN ^[^^16^^]^. PTEN expression was downregulated in tumor samples from a patient with gefitinib-refractory NSCLC ^[^^16^^]^. A study that used C4-2 cells, a prostate cancer cell line, showed that PTEN expression sensitized C4-2 cells to EGF and serum stimulation ^[^^17^^]^. Interestingly, this hypersensitivity to EGF stimulation was correlated with the amount of expressed PTEN. The restoration of PTEN expression alters the sensitivity of prostate cancer cells to EGFR inhibitors. There is also clinical evidence of the association of PTEN with prolonged survival after gefitinib treatment in EGFR-mutated lung cancer patients ^[^^18^^]^. Therefore, PTEN expression has therapeutic importance when using EGFR-targeted drugs for the treatment of NSCLC.

### Acquired resistance

The term acquired resistance (also known as secondary resistance) is used to describe patients who: (1) have been treated with a single-agent EGFR-TKI (e.g., gefitinib or erlotinib), (2) have either or both a tumor with an EGFR mutation associated with drug sensitivity or objective clinical benefit from treatment with an EGFR TKI, (3) suffer from systemic progression of disease (according to the Response Evaluation Criteria in Solid Tumors or World Health Organization) while on continuous treatment with gefitinib or erlotinib within the last 30 days, and (4) have not treated with intervening systemic therapy between the cessation of gefitinib or erlotinib and initiation of new therapy ^[^^19^^]^. In other words, secondary resistance affects patients who have initially responded to treatment, but subsequently stopped responding ^[^^10^^]^. Some of the molecular mechanisms of acquired resistance include T790M second mutation, mesenchymal epithelial transition factor (MET) amplification, ATP-binding cassette (ABC)-G2 transporter (ABCG2) and hepatocyte growth factor (HGF) overexpression, insulin-like growth factor (IGF) binding protein-3 (IGFBP3) downregulation, as well as ERBB3 activation.

#### T790M mutation

The T790M second mutation accounts for half of all resistances to gefitinib and erlotinib ^[^^20^^]^. This mutation is the substitution of threonine 790 with methionine. Threonine 790 is a “gatekeeper” residue, an important determinant of inhibitor specificity in the ATP binding pocket. Increased ATP affinity is the primary mechanism by which the T790M mutation confers drug resistance ^[^^20^^]^. This second mutation has been thought to cause resistance by sterically blocking the binding of TKIs ^[^^20^^]^. However, the T790M mutation may exist in only a small fraction of tumor cells before drug treatment, and the tumor cells harboring this mutation may be enriched over time during treatment with gefitinib or erlotinib ^[^^21^^]^.

#### MET amplification

MET, also known as hepatocyte growth factor receptor, is a proto-oncogenic receptor TK. MET amplification was detected in up to 20% of NSCLC with EGFR mutations progressing after an initial response to TKI therapy ^[^^22^^]^.

MET amplification activates ERBB3/PI3K/AKT signaling in EGFR-mutant lung cancers and causes resistance to EGFR TKIs ^[^^23^^]^. Engelman et al. ^[^^24^^]^ examined whether MET inhibition suppressed the growth of gefitinib-resistant (GR) lung cancer cells ^[^^24^^]^. They exposed HCC827-GR (GR NSCLC) cells to PHA-665752, a MET TKI, alone or in combination with gefitinib. The combined treatment resulted in substantial growth inhibition and induced apoptosis. In clinical settings, the development of anti-MET therapeutic strategies should be focused on patients with acquired EGFR-TKI resistance ^[^^22^^]^.

The correlation between MET amplification and T790M mutation is another interesting issue. Bean et al. ^[^^25^^]^ used array-based comparative genomic hybridization to compare genomic profiles of EGFR-mutant tumors from untreated patients with those from patients with acquired resistance^[^^25^^]^. Among 10 resistant tumors obtained from 9 patients with MET amplification, only 4 harbored the EGFR-T790M mutation. They also found that the EGFR-mutant lung adenocarcinoma cell line NCI-H820 harbors MET amplification in addition to a drug-sensitive EGFR mutation and the T790M mutation. Hence, MET amplification occurs independently of the T790M mutation. Another study indicated the presence of MET amplification in untreated NSCLC, and that MET amplification can increase MET TK activity in NSCLC ^[^^26^^]^.

#### ABCG2

In many cancers, the involvement of drug efflux out of cancerous cytoplasm has been confirmed to be related to acquired resistance to cytotoxic chemotherapy. ABC transporters, including ABCG2, are capable of pumping out various agents using ATP hydrolysis energy. Bessho et al. ^[^^27^^]^ examined the mechanism of resistance to 7-ethyl-10-hydroxycamptothecin (SN-38) in lung cancer by continuously exposing the NSCLC cell line NCI-H23 to SN-38 and selecting the SN-38-resistant clone H23/SN-38. Both the gene and protein expression of ABCG2/BCRP (ABCG2) in H23/SN-38 cells increased compared with those in NCI-H23 cells. The ABCG2-transduced human lung cancer PC-9 (PC-9/ABCG2) cells showed gefitinib resistance evidenced by their lower accumulation and higher efflux of gefitinib than their parental cells ^[^^28^^]^. Interestingly, the low concentration of gefitinib also inhibited ABCG2-dependent active drug extrusion and significantly reduced drug resistance in ABCG2-expressing cells ^[^^29^^]^.

#### HGF overexpression

Yano et al. ^[^^30^^]^ found that HGF induced gefitinib resistance in PC-9 and HCC827, two lung adenocarcinoma cell lines with EGFR-activating mutations. They first confirmed the presence of activating mutations in EGFR in human lung adenocarcinoma cell lines by direct sequencing, finding that PC-9 and HCC827 had deletions in exon 19. By applying HGF to these cells, they induced resistance of PC-9 and HCC827 cells to gefitinib in a dose-dependent manner. The effect of HGF was abrogated by the pretreatment of HGF with anti-HGF neutralizing antibody. A strategy to overcome HGF-induced gefitinib resistance has been studied using HCC827 cells engineered to stably express HCC827-HGF cells ^[^^31^^]^. HCC827-HGF cells exhibited resistance to gefitinib *in vitro* to an extent similar to that of HCC827-GR cells. Gefitinib combined with TAK-701, a humanized monoclonal antibody to HGF, inhibited the phosphorylation of MET, EGFR, extracellular signal-regulated kinase, and AKT in HCC827-HGF cells, resulting in the suppression of cell growth and indicating that autocrine HGF-MET signaling contributed to gefitinib resistance in these cells. The combination therapy of TAK-701 and gefitinib also markedly inhibited the growth of HCC827-HGF tumors *in vivo* ^[^^32^^]^.

#### IGFBP3 downregulation

IGFBP-3 was traditionally identified by its role as a binding protein as well as its association with IGF delivery and availability. IGFBP-3 has IGF-independent roles in inhibiting cell proliferation in cancer cell lines ^[^^32^^]^. Guix et al. ^[^^33^^]^ investigated the mechanisms of acquired resistance to the EGFR-TKI gefitinib by generating GR A431 squamous cancer cells ^[^^33^^]^. Gene expression analyses revealed that GR cells exhibited markedly reduced IGFBP-3 and IGFBP-4 RNA. The addition of recombinant IGFBP-3 restored the ability of gefitinib to downregulate PI3K/AKT signaling and inhibit cell growth.

#### ERBB3 activation

ERBB3/HER3 is one of the four members of the human EGFR/HER or ERBB receptor TK family. ERBB3 is gaining attention because of its recently appreciated role in the resistance of tumor cells to EGFR/ERBB2-targeted therapies ^[^^34^^]^. ERBB3 is a critical activator of PI3K signaling in EGFR (ERBB1)-, ERBB2 (HER2)-, and MET-addicted cancers. The reactivation of ERBB3 is a prominent way by which cancers become resistant to ERBB inhibitors ^[^^35^^]^. Heregulin can bind to and induce the activation of ERBB3. In one study, an EGFR mutant lung cancer cell line (HCC827) was rendered resistant to gefitinib by exogenous heregulin. This GR HCC827 cell line was re-sensitized by MM-121, an antibody against ERBB3. However, efforts to inactivate ERBB3 therapeutically in parallel with other ERBB receptors are challenging because its intracellular kinase domain is believed to be an inactive pseudokinase that lacks several key conserved (and catalytically important) residues, including the catalytic base aspartate ^[^^34^^]^.

## Conclusion

Our current perspectives on EGFR activating mutations have guided the determination of NSCLC patients who would benefit most from gefitinib or erlotinib treatment. Unfortunately, the inevitable occurrence of relapse in NSCLC patients has urged the further pursuance of oncology studies via both molecular biology and clinical trials for the future of NSCLC EGFR-TKI targeted therapy.

The following crucial agenda should be considered: 1) implementation of EGFR genotyping for lung adenocarcinoma, 2) development of a distinct management paradigm for oncogene-addicted cancers, 3) better utilization of rebiopsy tissue for molecular studies of resistance, and 4) genotype-guided clinical trials of targeted therapies for patients with acquired TKI resistance ^[^^36^^]^.
